# Association of Metabolic Syndrome and Albuminuria with Cardiovascular Risk in Occupational Drivers

**DOI:** 10.3390/ijms141121997

**Published:** 2013-11-06

**Authors:** Szu-Chia Chen, Jer-Ming Chang, Ming-Yen Lin, Meng-Ling Hou, Jer-Chia Tsai, Shang-Jyh Hwang, Hung-Chun Chen

**Affiliations:** 1 Division of Nephrology, Department of Internal Medicine, Kaohsiung Medical University Hospital, Kaohsiung Medical University, Kaohsiung 807, Taiwan; E-Mails: scarchenone@yahoo.com.tw (S.-C.C.); jemich@kmu.edu.tw (J.-M.C.); mingyenlin3@gmail.com (M.-Y.L.); sjhwang@kmu.edu.tw (S.-J.H.); chenhc@kmu.edu.tw (H.-C.C.); 2 Department of Internal Medicine, Kaohsiung Municipal Hsiao-Kang Hospital, Kaohsiung Medical University, Kaohsiung 812, Taiwan; 3 Faculty of Medicine, Kaohsiung Medical University, Kaohsiung 807, Taiwan; 4 Faculty of Renal Care, College of Medicine, Kaohsiung Medical University, Kaohsiung 807, Taiwan; 5 Department of Nutrition, Kaohsiung Municipal Hsiao-Kang Hospital, Kaohsiung Medical University, Kaohsiung 812, Taiwan; E-Mail: 0900033@kmhk.org.tw

**Keywords:** occupational driver, metabolic syndrome, albuminuria, Framingham Risk Score

## Abstract

**Background and Aim:**

Metabolic syndrome (MetS) and albuminuria increase cardiovascular risk. However, in occupational drivers, the clinical significance of albuminuria and its association with MetS remain unclear. We investigated the prevalence of MetS, albuminuria and cardiovascular risk, and its associated risk factors in occupational drivers;

**Methods:**

441 occupational drivers and 432 age- and sex-stratified matched counterpart controls were enrolled. MetS was defined using Adult Treatment Panel III for Asians. Albuminuria was defined as urine albumin-to-creatinine ratio ≥ 30 mg/g. Cardiovascular disease risk was evaluated by Framingham Risk Score (FRS);

**Results:**

A significantly higher prevalence of MetS (43.1% *vs.* 25.5%, *p* < 0.001), albuminuria (12.0% *vs.* 5.6%, *p* = 0.001) and high FRS risk ≥ 10% of 10-year risk (46.9% *vs.* 35.2%, *p* < 0.001) was found in occupational drivers compared with their counterpart controls. Multiple logistic regression analysis showed that old age, a history of diabetes, gout and betel nut chewing, less exercise and albuminuria (odds ratio [OR], 2.75; *p* = 0.01) were risk factors for MetS, while a history of renal disease, diabetes and hypertension, and MetS (OR, 2.28; *p* = 0.01) were risk factors for albuminuria in occupational drivers;

**Conclusions:**

Our study demonstrated that MetS and albuminuria were public health problems in occupational drivers. An education program for promoting healthy lifestyle and a regular occupational health visit for early detection and interventions should be established.

## 1. Introduction

Metabolic syndrome (MetS), which comprises central obesity, dyslipidemia, high blood pressure (BP) and impaired fasting glucose, is a known risk factor for albuminuria and cardiovascular disease [1,2]. On the other hand, albuminuria is associated with insulin resistance, diabetes mellitus (DM) and the components of MetS, such as hypertension, obesity and dyslipidemia [3]. Besides, albuminuria increases the risk of cardiovascular disease [4]. The Framingham Risk Score (FRS) allows clinicians to estimate the individual patient risk of coronary heart disease (stable angina, unstable angina, myocardial infarction, and cardiovascular death) by using traditional cardiac risk factors including age, gender, systolic BP, treatment of hypertension, total cholesterol, high-density lipoprotein (HDL) cholesterol, and cigarette smoking [5]. Most previous studies of the relationship between MetS and albuminuria have been conducted in the general population [6,7], and little is known about the impact in occupational drivers, a population with high prevalence of MetS [8].

Occupational health of drivers has great impact on public safety [9]. Occupational drivers have been reported to have higher health risks in MetS, undiagnosed DM, and cardiovascular diseases, low back pain, sleep disorders, obesity and peptic ulcer [8,10–15]. Some epidemiological studies have identified several important risk factors for these disorders, including life-style risk factors (*i.e.*, alcohol consumption, cigarette smoking, lack of physical activity, unhealthy diet) and psychological reason (*i.e.*, high stress due to hazardous working conditions, irregular sleep habits, *etc.*) [8,10–16]. However, it is still uncertain whether albuminuria plays a role in the development of MetS and cardiovascular disease in occupational drivers. Additionally, the factors for MetS, albuminuria and cardiovascular risk in this population need to be identified. Accordingly, the aims of this study were to investigate the prevalence of MetS, albuminuria and cardiovascular risk and its associated risk factors in occupational drivers.

## 2. Results

**Figure 1:**
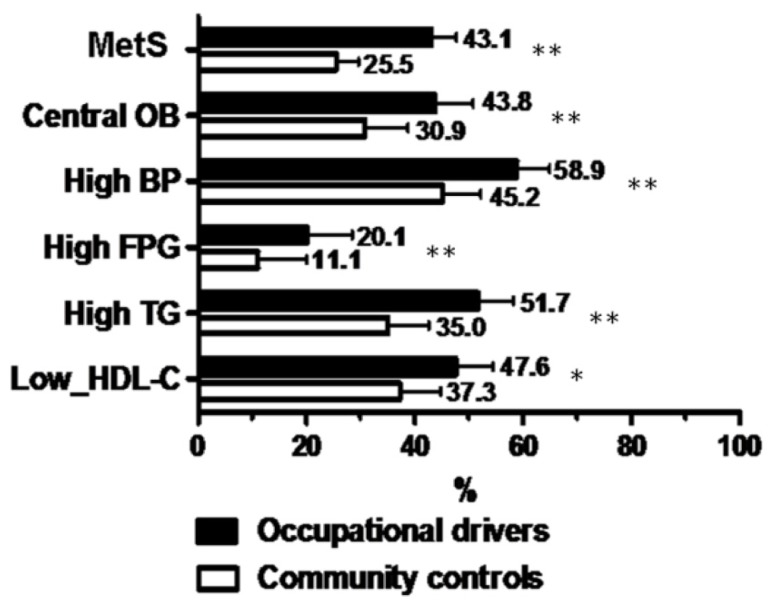
Prevalence of metabolic syndrome (MetS) and its components in occupational drivers and community controls. OB: obesity; BP: blood pressure; FPG: fasting plasma glucose; TG: triglycerides; HDL-C: high-density lipoprotein cholesterol; ** *p* < 0.001, * *p* < 0.01.

The mean ages of the 441 occupational drivers and 432 controls were 46.5 ± 9.4 and 47.5 ± 13.6 years respectively. The differences between occupational drivers and community controls are shown in Table 1. Compared with community controls, occupational drivers were associated with lower class of education, lower prevalence of a history of hypertension, higher percentages of smoking, alcohol drinking, and betel nut chewing, lower percentage of exercise habit, higher systolic and diastolic BP, higher body mass index, wider waist circumference, higher fasting glucose, higher total cholesterol, lower HDL-cholesterol and higher triglyceride. In addition, although the prevalence of a history of hypertension was significantly lower than that of controls (10.0% *vs.* 15.0%; *p* = 0.01), the prevalences of MetS (43.1% *vs.* 25.5%, *p* < 0.001), albuminuria (12.0% *vs.* 5.6%, *p* = 0.01) and high FRS risk (46.9% *vs.* 35.2%, *p* < 0.001) were significantly higher in occupational drivers compared with controls. The prevalence of MetS and its components in occupational drivers and community controls is shown in Figure 1.

### 2.1. Determinants of MetS in All Subjects

Table 2 shows the determinants of MetS in all subjects. In the univariate regression analysis, MetS was found to be significantly associated with occupational drivers, old age, males, a history of DM and gout, a habit of smoking (current *vs.* former), and consumption of alcohol or betel nut (ever *vs.* never) and albuminuria. In the multivariate forward analysis, occupational drivers (*vs.* community controls; odds ratio [OR], 1.70; 95% confidence interval [CI], 1.20 to 2.42; *p* = 0.01), old age (OR, 1.03; 95% CI, 1.01 to 1.04; *p* < 0.001), males (OR, 4.92; 95% CI, 1.43 to 16.96; *p* = 0.01), a history of DM (OR, 2.60; 95% CI, 1.35 to 5.00; *p* = 0.01) and gout (OR, 2.31; 95% CI, 1.26 to 4.23; *p* = 0.01), betel nut chewing (OR, 2.03; 95% CI, 1.41 to 2.92; *p* < 0.001) and albuminuria (OR, 2.75; 95% CI, 1.63 to 4.65; *p* < 0.001) were independently associated with MetS.

### 2.2. Determinants of Albuminuria in All Subjects

Table 3 displays a logistic regression analysis for albuminuria in all subjects. In the univariate regression analysis, albuminuria was found to be significantly associated with occupational drivers, old age, a history of renal disease, DM, hypertension and gout, and MetS. In the multivariate forward analysis, occupational drivers (*vs.* community controls; OR, 2.65; 95% CI, 1.51 to 4.87; *p* = 0.01), old age (OR, 1.05; 95% CI, 1.02 to 1.08; *p* < 0.001), a history of renal disease (OR, 2.68; 95% CI, 1.14 to 6.30; *p* = 0.02), and hypertension (OR, 2.40; 95% CI, 1.32 to 4.36; *p* = 0.01) and MetS (OR, 2.58; 95% CI, 1.56 to 4.29; *p* < 0.001) were independently associated with albuminuria.

We further performed stratification analysis on patients without diabetes (*n* = 805), and found that the occupational drivers (*vs.* community controls) were still a risk factor for MetS (OR, 1.60; 95% CI, 1.12 to 2.28; *p* = 0.01) and albuminuria (OR, 2.18; 95% CI, 1.21 to 3.93; *p* = 0.01) after multiple adjustment (in the model for MetS: covariates included age, sex, a history of gout, a habit of smoking and consumption of alcohol or betel nut and albuminuria; in the model for albuminuria: covariates included age, sex, a history of renal disease, hypertension and gout and MetS).

**Figure 2:**
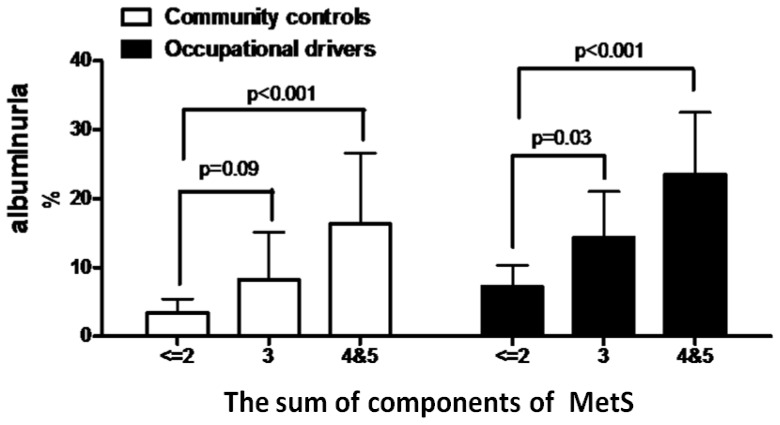
Prevalence of albuminuria according to the sum of components of metabolic syndrome in occupational drivers and community control.

In all subjects, compared with subjects without MetS, subjects with MetS had higher prevalence of albuminuria (16.0% *vs.* 5.1%, *p* < 0.001). Figure 2 reveals the prevalence of albuminuria according to the sum of components of MetS in occupational drivers and community controls.

### 2.3. Determinants of MetS and Albuminuria in Occupational Drivers

We performed two multivariate forward analyses in occupational drivers (Table 4). In the first multivariate analysis (the model for MetS: covariates including age, sex, a history of DM and gout, betel nut chewing, exercise habit and albuminuria), old age (OR, 1.03; 95% CI, 1.00 to 1.05; *p* = 0.03), a history of DM (OR, 6.29; 95% CI, 1.64 to 224.04; *p* = 0.01) and gout (OR, 3.25; 95% CI, 1.35 to 7.82; *p* = 0.03), betel nut chewing (OR, 2.06; 95% CI, 1.36 to 3.14; *p* = 0.01), less exercise (OR, 0.55; 95% CI, 0.34 to 0.90; *p* = 0.02) and albuminuria (OR, 2.75; 95% CI, 1.41 to 5.37; *p* = 0.01) were independently associated with MetS. In the second multivariate analysis (the model for albuminuria: covariates including age, sex, a history of renal disease, DM and hypertension and MetS), a history of renal disease (OR, 4.16; 95% CI, 1.48 to 11.73; *p* = 0.01), DM (OR, 4.98; 95% CI, 1.79 to 13.87; *p* = 0.01) and hypertension (OR, 3.66; 95% CI, 1.69 to 7.92; *p* = 0.01) and MetS (OR, 2.28; 95% CI, 1.20 to 4.30; *p* = 0.01) were independently associated with albuminuria.

**Figure 3:**
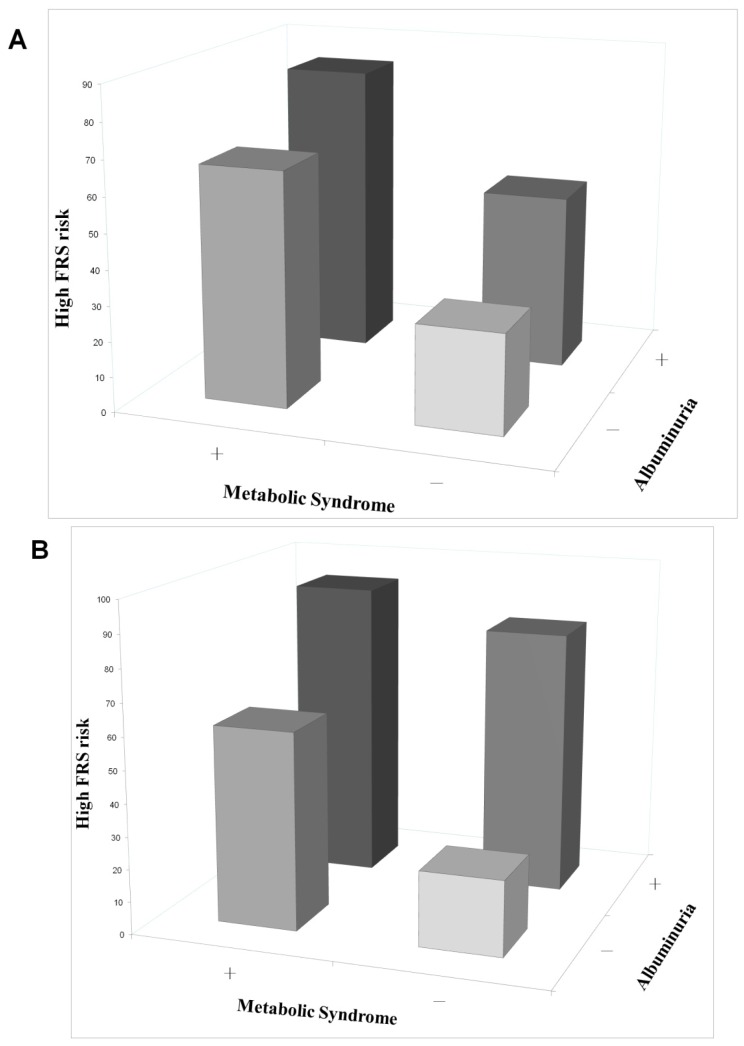
Occupational drivers (**A**) and community controls (**B**) with both metabolic syndrome and albuminuria showed the highest rate for high FRS risk ≥ 10% of 10-year risk.

We divided occupational drivers into four groups according to the presence of MetS and albuminuria. The high FRS risk rate in the four groups was 28.3% (without MetS and albuminuria), 66.5% (with MetS, but without albuminuria), 50.0% (without MetS, but with albuminuria), and 82.9% (with MetS and albuminuria) (*p* < 0.001), respectively (Figure 3A). Besides, the high FRS risk rate in the four groups, in the control group it was 23.2% (without MetS and albuminuria), 60.8% (with MetS, but without albuminuria), 81.8% (without MetS, but with albuminuria), and 92.3% (with MetS and albuminuria) (*p* < 0.001), respectively (Figure 3B). The correlation between MetS, albuminuria, and FRS risk in the control group was similar to that observed in the occupational driver group.

## 3. Discussion

In the present study, there was a significantly higher prevalence of MetS, albuminuria and high FRS risk in occupational drivers compared with their counterpart controls. MetS was significantly associated with albuminuria, and MetS and albuminuria might have an effect on cardiovascular risk in occupational drivers.

Previous studies have reported that occupational drivers had higher prevalence of MetS (24.0% to 35.9%) [9,12,17–19]. Occupational drivers are particularly at risk of excessive MetS due to substantial changes in lifestyle habits (*i.e.*, alcohol consumption, cigarette smoking, betel nut chewing, less exercise), poor working environment (*i.e.*, irregular and long working hours, unhealthy diet, working in a fixed position, physical inactivity) and psychological reasons (*i.e.*, being highly stressed due to the hazardous working conditions, having irregular sleep habits) [8,10–16,19,20]. In our study, the majority of occupational drivers had irregular exercise habit (75.3%) and ate out ≥2 times/day (71.7%). Besides, they had higher percentages of smoking, alcohol use and betel nut chewing habits than that of the controls. Individuals with MetS had a higher probability of developing cardiovascular disease because each component of MetS is a known factor for the development of cardiovascular disease [1,21]. Therefore, the high prevalence of MetS in occupational drivers is a public health concern, and health care measurements for them should be taken to prevent and detect MetS and cardiovascular disease.

One important finding of our study was that occupational drivers had increased risk for albuminuria, and albuminuria was associated with MetS. Moreover, our study also found MetS and albuminuria were cardiovascular risk factors in occupational drivers and community controls. We found that the high FRS risk rate in the groups with MetS and albuminuria in occupational drivers and community controls. Clinically, albuminuria is associated with hypertension, obesity, insulin resistance, DM and MetS [3,4]. Endothelial dysfunction is likely to be involved in the initiation and development of albuminuria, followed by the development and progression of atherosclerosis, further resulting in adverse cardiovascular outcomes [4,22]. In our study, although occupational drivers had a similar DM prevalence and lower prevalence of a history of hypertension than that of the controls, occupational drivers still had a higher prevalence of albuminuria and high FRS risk. The reason for the positive association between occupational drivers with albuminuria and cardiovascular risk might be related to high BP, obesity, high fasting glucose, dyslipidemia (higher total cholesterol, lower HDL-cholesterol and high triglyceride) and similar factors associated with MetS in occupational drivers (*i.e.*, unhealthy lifestyle habits, poor working environment, psychological reasons). In conclusion, a rather excessive prevalence of cardiovascular risk factors in occupational drivers was observed. Specific attention to albuminuria might be necessary in occupational drivers especially when they simultaneously have MetS.

The FRS is the most commonly used coronary heart disease risk prediction instrument in clinical settings by counting traditional cardiac risk factors including age, sex, systolic BP, treatment of hypertension, total cholesterol, HDL-cholesterol and cigarette smoking [5]. FRS is used to quantify 10-year risk. In our study, we found that occupational drivers had a “high” risk categorized by FRS (≥10% of 10-year risk of coronary heart disease). Therefore, occupational drivers may need intensive medical risk reduction.

Another important finding of our study was the identification of betel nut use as a risk factor for MetS in occupational drivers. Betel nut use, the fourth most addictive habit in the world, occurs in about 10% of the world population, including that of Taiwan [23]. Previous studies have found that chewing betel nut is associated with MetS due to sympathetic activation, central obesity, insulin resistance, oxidative stress and inflammation [24–27]. Regular screening for betel nut chewing may help prevent MetS development, and an anti-betel nut-chewing program is warranted for occupational drivers.

The prevalence of a history of DM, hypertension and cardiovascular disease was lower in occupational drivers than in community controls. However, systolic BP, diastolic BP and fasting glucose was higher in occupational drivers than in community controls. The reason for the inconsistent association might be related to the lack of awareness and careless attitude toward disease in occupational drivers and inconsistent personal history taking due to different questionnaires from survey workers. Although there appears to be a low prevalence of a history of DM, hypertension, and cardiovascular disease in occupational drivers, occupational drivers still have a higher prevalence of MetS, albuminuria and high FRS risk. This implies that occupational driving itself might be a risk factor of MetS, albuminuria and high FRS risk independent of the other mentioned factors.

Several limitations of our study need to be addressed. First, the diagnosis of albuminuria was based on a single laboratory measurement; however, this drawback was minimized by the accurate assays of urinary albumin and creatinine as described in the Methods section. In addition, this study was cross-sectional, so a causal relationship and long-term clinical outcomes could not be confirmed. Finally, this screening study was conducted locally and with limited numbers of participants, and thus the generalization potential of our results is limited.

## 4. Subjects and Methods

### 4.1. Study Patients and Design

**Figure 4:**
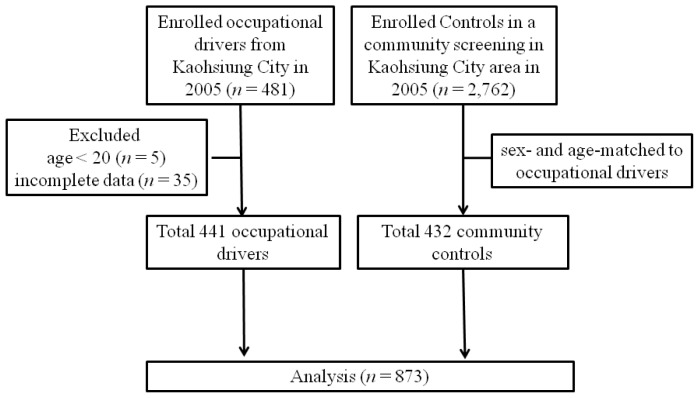
A flowchart of participant enrollment.

We enrolled 481 occupational drivers voluntarily from vocational associations in the Kaohsiung City (Taiwan) area in 2005. To calculate FRS, we excluded patients with incomplete data (*n* = 35), and those younger than 20 years of age (*n* = 5). Finally, a total of 441 occupation drivers were included, including truck drivers (*n* = 275; 62.4%), taxi drivers (*n* = 101; 22.9%), trailer drivers (*n* = 60; 14.6%), and bus drivers (*n* = 5; 1.1%). Controls were selected voluntarily from the population participating in a community screening program that consisted of 2762 participants in the Kaohsiung City area in 2005 (men/women, 771/1991; mean age, 52.5 ± 13.2 years). In selecting controls for occupational drivers, 432 sex- and age-stratified matched persons were randomly selected as controls (Figure 4). The study was approved by the Institutional Review Board (Kaohsiung, Taiwan) and conducted according to the principles expressed in the Declaration of Helsinki. Informed consent was obtained from all study participants and controls.

### 4.2. Laboratory Investigations

Biochemical data were measured from fasting blood samples overnight for at least 8 or 10 h using an autoanalyzer (COBAS Integra 400 plus; Roche Diagnostics, www.roche.com/diagnostics/). Serum creatinine was measured by the compensated Jaffé (kinetic alkaline picrate) method in a Roche/Integra 400 Analyzer (Roche Diagnostics, Mannheim, Germany) using a calibrator traceable to isotope-dilution mass spectrometry [28]. The value of estimated glomerular filtration rate was calculated using the 4-variable equation in the modification of diet in renal disease study [29]. Urinary albumin and creatinine were measured from 1-spot urine sample using the same analyzer. Intra-assay coefficients of variation for urinary albumin and creatinine were 1.09% and 0.74% respectively. Interassay coefficients of variation for urinary albumin and creatinine were 1.48% and 0.94% respectively.

### 4.3. Definition of MetS and Albuminuria

MetS was defined as having 3 of the following 5 abnormalities based on the standard of the National Cholesterol Education Program Adult Treatment Panel III [30] and modified criteria for Asians [31]: (1) abdominal obesity, defined as waist circumference > 90 cm for men and > 80 cm for women; (2) hypertriglyceridemia, defined as triglyceride concentration ≥ 150 mg/dL; (3) low HDL-cholesterol concentration, defined as HDL-cholesterol concentration < 40 mg/dL in men and < 50 mg/dL in women; (4) hyperglycemia, defined as fasting whole-blood glucose concentration ≥ 110 mg/dL or DM; and (5) high BP, defined as systolic BP ≥ 130 mm Hg, diastolic BP ≥ 85 mm Hg, or physician-diagnosed or -treated hypertension. Albuminuria was defined as urinary albumin-creatinine ratio ≥ 30 mg/g.

### 4.4. FRS Calculation and Risk Category

The FRS was calculated based on a model comprised of age, gender, total cholesterol, HDL-cholesterol, systolic and diastolic BP, treatment of DM and cigarette smoking [32]. FRS identified individuals categorically as “low” (<10% 10-year risk), or “high” risk (≥10% risk).

### 4.5. Questionnaire

All study participants completed a questionnaire survey to provide information about sociodemographic data, personal history of kidney diseases, DM, hypertension, liver disease, cardiovascular disease, dyslipidemia and gout, and lifestyle regarding smoking (current *vs.* former) and consumption habits of alcohol or betel nut (ever *vs.* never), and exercise habits (achieving the goal of 30 min/time and 3 times/week).

### 4.6. Statistical Analysis

Statistical analysis was performed using SPSS 17.0 for windows (SPSS Inc. Chicago, IL, USA). Data were expressed as percentages, mean ± standard deviation or median (25th–75th percentile) for triglyceride. The differences between groups were checked by Chi-square test for categorical variables or by independent *t*-test for continuous variables. Multiple comparisons among the study groups were performed by one-way analysis of variance (ANOVA). Multiple logistic regression analysis with forward selection approach was used to identify the factors associated with MetS, and albuminuria. Age, sex, and significant variables in univariate analysis were selected for multivariate analysis. A difference was considered significant if the *p* value was less than 0.05.

## 5. Conclusions

Our results demonstrate a noteworthy higher prevalence of MetS, albuminuria and high cardiovascular risk in occupational drivers compared to controls. In addition, MetS and albuminuria might have an effect on the cardiovascular risk in this population. Therefore, it is crucial for public safety to identify occupational drivers with MetS and albuminuria for aggressive treatment interventions. A regular occupational health visit should be established for early detection and intervention to lower cardiovascular risk.

## Figures and Tables

**Table 1:** Comparison of baseline characteristics between occupational drivers and community controls.

Variables	Occupational drivers (*n* = 441)	Community controls (*n* = 432)	*p* value
Age (year)	46.5 ± 9.4	47.5 ± 13.6	0.21
Male gender (%)	96.8	95.8	0.44

**Education (year)**			

≤6	17.5	11.1	<0.001
7–12	73.7	20.1	–
≥12	8.8	63.4	–
Unknown	0	5.4	–

**Driving time (h/days)**			

≤8	38.3	–	–
9–12	50.3	–	–
≥13	11.3	–	–

**Co-morbidities**			

Renal diseases (%)	4.8	3.2	0.32
Diabetes mellitus (%)	4.3	6.7	0.08
Hypertension (%)	10.0	15.0	0.01
Liver diseases (%)	4.3	5.1	0.48

Cardiovascular disease (%)	2.7	4.9	0.07

Dyslipidemia (%)	4.1	6.3	0.11
Gout (%)	6.6	5.3	0.55

**Habits**			

Smoking (current) (%)	47.2	0	<0.001
Drinking (ever) (%)	71.2	41.0	<0.001
Betel nut chewing (ever) (%)	50.6	6.5	<0.001
Exercise (30 min/time, 3 times/week) (%)	24.7	46.1	<0.001
Systolic BP (mmHg)	133.0 ± 19.3	125.9 ± 16.6	<0.001
Diastolic BP (mmHg)	82.8 ± 12.6	79.2 ± 10.8	<0.001
Body mass index (kg/m^2^)	26.2 ± 3.6	24.5 ± 3.3	<0.001
Waist circumference (cm)	88.7 ± 10.3	85.7 ± 9.1	<0.001

**Laboratory parameters**			

Glucose (mg/dL)	105.4 ± 47.3	92.1 ± 28.1	<0.001
Total cholesterol (mg/dL)	211.4 ± 47.5	201.6 ± 37.2	0.01
HDL-cholesterol (mg/dL)	53.2 ± 13.6	55.3 ± 13.6	0.02
LDL-cholesterol (mg/dL)	133.3 ± 39.2	132.0 ± 33.9	0.65
Triglyceride (mg/dL)	152 (105.25–229.1)	118.55 (84.2–178.25)	<0.001
Uric acid (mg/dL)	6.8 ± 1.5	6.7 ± 1.4	0.29
eGFR (mL/min/1.73 m^2^)	80.6 ± 15.6	79.9 ± 14.9	0.49
MetS (%)	43.1	25.5	<0.001
Albuminuria (%)	12.0	5.6	0.01
High FRS risk (%)	46.9	35.2	<0.001

Abbreviations: BP: blood pressure; HDL: high density lipoprotein; LDL: low density lipoprotein; MetS: metabolic syndrome; FRS: Framingham Risk Score; The FRS is used to identify individuals categorically as “low” (<10% of 10-year risk), or “high” risk (≥10% risk).

**Table 2:** Determinants of MetS in all subjects.

Parameter	Univariate		Multivariate (Forward)	
	
	OR (95% CI)	*p*	OR (95% CI)	*p*
Occupational drivers *vs.* Community controls	2.22 (1.66–2.95)	<0.001	1.70 (1.20–2.42)	0.01
Age (per 1 year)	1.02 (1.01–1.04)	<0.001	1.03 (1.01–1.04)	<0.001
Male gender	5.28 (1.59–17.47)	0.01	4.92 (1.43–16.96)	0.01

**Driving time (h/days)**				

≤8	Reference	–	–	–
9–12	1.01 (0.67–1.51)	0.97	–	–
≥13	1.15 (0.61–2.16)	0.67	–	–

**Co-morbidities**				

Renal diseases	1.84 (0.93–3.62)	0.08	–	–
Diabetes mellitus	2.82 (1.56–5.10)	0.01	2.60 (1.35–5.00)	0.01
Hypertension	1.45 (0.96–2.19)	0.08	–	–
Liver diseases	0.77 (0.39–1.54)	0.46	–	–
Cardiovascular disease	0.82 (0.38–1.74)	0.60	–	–
Dyslipidemia	1.41 (0.77–2.59)	0.27	–	–
Gout	3.00 (1.69–5.33)	<0.001	2.31 (1.26–4.23)	0.01

**Habits**				

Smoking (current *vs.* former)	2.07 (1.50–2.85)	<0.001	–	–
Drinking (ever *vs.* never)	1.36 (1.02–1.81)	0.04	–	–
Betel nut chewing (ever *vs.* never)	2.39 (1.76–3.23)	<0.001	2.03 (1.41–2.92)	<0.001
Exercise (30 min/time, 3 times/week)	0.75 (0.56–1.01)	0.06	–	–
Albuminuria	3.57 (2.20–5.80)	<0.001	2.75 (1.63–4.65)	<0.001

Values expressed as odds ratio (OR) and 95% confidence interval (CI).

**Table 3:** Determinants of albuminuria in all subjects.

Parameter	Univariate		Multivariate (Forward)	
	
	OR (95% CI)	*p*	OR (95% CI)	*p*
Occupational drivers *vs.* Community controls	2.32 (1.41–3.84)	0.01	2.65 (1.51–4.87)	0.01
Age (per 1 year)	1.05 (1.03–1.08)	<0.001	1.05 (1.02–1.08)	< 0.001
Male gender	0.51 (0.19–1.35)	0.17	–	–

**Driving time (h/days)**				

≤8	Reference	–	–	–
9–12	0.77 (0.42–1.42)	0.40	–	–
≥13	0.87 (0.33–2.26)	0.77	–	–

**Co-morbidities**				

Renal diseases	3.88 (1.75–8.62)	0.01	2.68 (1.14–6.30)	0.02
Diabetes mellitus	3.39 (1.65–1.95)	0.01	–	–
Hypertension	2.99 (1.73–5.18)	<0.001	2.40 (1.32–4.36)	0.01
Liver diseases	1.11 (0.39–3.21)	0.85	–	–
Cardiovascular disease	1.88 (0.71–5.03)	0.21	–	–
Dyslipidemia	1.62 (0.66–3.97)	0.29	–	–
Gout	2.29 (1.07–4.91)	0.03	–	–

**Habits**				

Smoking (current *vs.* former)	1.11 (0.65–1.90)	0.70	–	–
Drinking (ever *vs.* never)	1.00 (0.63–1.61)	0.99	–	–
Betel nut chewing (ever *vs.* never)	0.96 (0.57–1.62)	0.89	–	–
Exercise (30 min/time, 3 times/week)	1.00 (0.61–1.63)	0.99	–	–
MetS	3.57 (2.20–5.80)	<0.001	2.58 (1.56–4.29)	<0.001

Values expressed as odds ratio (OR) and 95% confidence interval (CI).

**Table 4:** Determinants of metabolic syndrome (MetS) and albuminuria in occupational drivers.

Parameter	Multivariate (Forward)	
**MetS**	**Adjusted OR (95% CI)**	***p***

Age (per 1 year)	1.03 (1.00–1.05)	0.03
Diabetes mellitus	6.29 (1.64–24.04)	0.01
Gout	3.25 (1.35–7.82)	0.03
Betel nut chewing (ever *vs.* never)	2.06 (1.36–3.14)	0.01
Exercise (30 min/time, 3 times/week)	0.55 (0.34–0.90)	0.02
Albuminuria	2.75 (1.41–5.37)	0.01

**Albuminuria**	**Adjusted OR (95% CI)**	***p***

Renal diseases	4.16 (1.48–11.73)	0.01
Diabetes mellitus	4.98 (1.79–13.87)	0.01
Hypertension	3.66 (1.69–7.92)	0.01
MetS	2.28 (1.20–4.30)	0.01

Values expressed as odds ratio (OR) and 95% confidence interval (CI); For metabolic syndrome: adjusted for age, sex, a history of diabetes mellitus, and gout, betel nut chewing, exercise, and albuminuria; For albuminuria: adjusted for age, sex, renal disease, a history of diabetes mellitus, and hypertension, and MetS.
